# MiR-429 Inhibits the Angiogenesis of Human Brain Microvascular Endothelial Cells through SNAI2-Mediated GSK-3*β*/*β*-Catenin Pathway

**DOI:** 10.1155/2021/6753926

**Published:** 2021-12-20

**Authors:** Yameng Sun, Shenghao Ding, Yiling Fan, Fei Shen, Qing Dong, Bing Zhao, Yaohua Pan, Jieqing Wan

**Affiliations:** ^1^Department of Neurology, Renji Hospital, Shanghai Jiaotong University School of Medicine, Shanghai 200127, China; ^2^Department of Neurosurgery, Renji Hospital, Shanghai Jiaotong University School of Medicine, Shanghai 200127, China; ^3^Department of Neurology, South Campus, Renji Hospital, Shanghai Jiaotong University School of Medicine, Shanghai 200127, China

## Abstract

MicroRNA (miRNA) dysfunction has been confirmed as a key event of ischemic stroke appearance. This study is aimed at revealing the role of miR-429 in the angiogenesis of HBMECs. The HBMECs were treated with oxygen and glucose deprivation (OGD) to establish the ischemic cell model. The qRT-PCR was used to measure the expression levels of the miR-429 in the serums of the patients or cells, and CCK-8, wound healing assay, and tube formation assay were used to observe the effects of miR-429 on the phenotype of HBMECs. Moreover, the Targetscan, dual-luciferase reporter assay, and Western blot were used to reveal the downstream target and regulation mechanism of miR-429 in OGD-induced HBMECs. The results showed that miR-429 was significantly upregulated in the serums of the patients, and overexpressed miR-429 could extremely inhibit the viability, migration, and tube formation of OGD-induced HBMECs. Furthermore, it was found that SNAI2 was a downstream factor of miR-429, and SNAI2 could rescue the effects of miR-429 on OGD-induced HBMECs. Besides, the Western blot showed that miR-429 could affect the activity of GSK-3*β*/*β*-catenin pathway via inhibiting the expression of SNAI2. In conclusion, this study suggests that miR-429 inhibits the angiogenesis of HBMECs through SNAI2-mediated GSK-3*β*/*β*-catenin pathway.

## 1. Introduction

Stroke is an acute cerebrovascular disease induced by the sudden rupture of blood vessels or vascular obstruction, which seriously threaten the health of millions of people all over the world in each year [1, 2]. More than 70% of stroke can contribute as ischemic type, and nearly 30% patients were related with primary hemorrhage [3]. For restoring the blood supply, the progression of the angiogenesis will undergo when the body suffered insufficient blood supply [4]. The study has indicated that the angiogenesis play an important role in the repair of cerebral ischemia, which may prevent the patients away from stroke [5]. Angiogenesis is a complicated and sequential process, which occurs in the ischemic penumbra within hours after stroke and lasts for weeks. The newly formed vessels after stroke not only improved tissue perfusion but also closely linked with neurovascular remodeling, axonal sprouting, and remyelination [1–4]. It is well established that angiogenesis is strongly associated with improvement of neurological deficits after stroke [3, 4]. Patients with higher blood vessel density showed better functional recovery after ischemic stroke impact. Therefore, improving the angiogenesis of patients has been increasingly recognized as a promising therapeutic strategy for the treatment of ischemic stroke.

MicroRNAs (miRNA), as noncoding single RNA with the length of approximate 20 nucleotides, have been confirmed as key factors in multiple activities of cells [6, 7]. In recent decade, increasing evidences have been confirmed that dysfunctions of miRNAs involve in the formation and development of many disease ranging from cancers to neurological diseases [8, 9]. MiRNAs are characterized by binding the 3′-UTR of special mRNA to regulate the expression of the related proteins [10, 11]. It has shown that miR-429 could inhibit the development of colorectal cancer via targeting in large tumor suppressor kinase (LATS2), and decreased miR-429 could promote the expression of SRY-box transcription factor 2 (SOX2) and B-cell lymphoma 2 (BCL2) to attenuate the neuronal injury induced by accumulation of amyloid *β*-protein [12].

SNAI2, an angiogenesis-related factor, was highly expressed under the high glucose condition and also led to viability and migration of human brain microvascular endothelial cells (HBMECs) [13]. It was also found that in this study, the weakened migration and viability of HBMECs could be abolished by increased SNAI2. Yu et al. have also pointed out that SNAI2 downregulation is related with reduced angiogenesis of mice with retinal neovascularization [14]. This study demonstrated that overexpression of miR-203 suppressed the angiogenesis in mice with pathological retinal neovascularization disease via the inactivation of GSK-3*β*/*β*-catenin pathway by inhibiting SNAI2.

In our study, we attempted to investigate the association of miR-429 with ischemic stroke and uncover the regulation mechanism of miR-429 underlying the progression of ischemic stroke.

## 2. Material and Methods

### 2.1. Cell Culture and OGD Treatment

Human BMECs purchased from Generay Biotech (Shanghai, China) were used for this study, and Dulbeco's Modified Eagle's Medium (DMEM) containing 10% fetal bovine serum (FBS) was used to culture cells. The cells were cultured in an 37°C incubator with 5% CO_2_, and the subculture of the cells were performed when confluence of the cells was at 90%.

The cells were treated with oxygen and glucose deprivation (OGD) for the establishment of ischemia model. In short, the cells were cultured with glucose-free DMEM and then transferred to an anaerobic chamber (with 5% and 95% N_2_) at 37 for 2 hours. After that, the cells were returned to normoxic culture condition for 0, 12, and 24 hours.

### 2.2. Cell Transfection

The negative control of miRNA (miR-NC), miR-429 mimics, or pcDNA-SNAI2 was designed and purified by Generay Biotech (Shanghai, China). The cells were seed in 6-well plates, and cell transfections were performed when confluence of the cells was at 70%. Briefly, 4 *μ*g of DNA, 100 pmol RNA, or 10 *μ*L Lipofectamine 2000 was diluted and incubated with 250 *μ*L serum-free medium for 5 min, respectively.

The diluted transfectants were coincubated with the equal volume of diluted Lipofectamine 2000 at 25°C for 20 min. Finally, the 500 *μ*L of mixtures were added in each well, and then, the cells were cultured for 24 hours.

### 2.3. qRT-PCR

TRIzol purchased from Generay Biotech (Shanghai, China) were utilized to extract total RNA extraction from cells, and the concentration of extracts was measured by ultraviolet spectrophotometry. A PrimeScript® RT reagent Kit (Thermo Fisher, Massachusetts, USA) was used for the reverse transcription of the total RNA. 1 *μ*L of the primers (10 *μ*mol/L) of miR-429 synthesized and purified by Synbio Technology (Suzhou, China) were configured the reaction system according to the instruction of KAPA qRT-PCR kit (Sigma-Aldrich, Missouri, USA). The primer sequences of U6 and miR-429 are shown in Table 1.

### 2.4. Western Blot

The RIPA buffer purchased from Generay Biotech (Shanghai, China) were added into the cells for the extraction of the total proteins, and the concentration of the total proteins was measured by BCA protein assay kit (Thermo Fisher, Massachusetts, USA). The related proteins were isolated from the total proteins by polyacrylamide gel and then were transferred on the PVDF membranes. Subsequently, the PVDF membranes were blocked by 5% fat-free milk for 1 hour and then incubated with the related primary antibodies at 4°C overnight. After that, the membranes were incubated with the related second antibodies at 25°C for 1 hour. Finally, the expression levels of the proteins were observed by a chemiluminescence detection system. The antibodies were used as follows: anti-*β*-catenin (1 : 500; Cell Signaling Technology), anti-p-GSK3*β* (at Ser9, 1 : 500; Cell Signaling Technology), anti-GSK3*β* (1 : 1000, ThermoFisher, Massachusetts, USA), and anti-SNAI2 (1 : 2000, ThermoFisher, Massachusetts, USA).

### 2.5. Dual-Luciferase Reporter Gene Assay

The pmirGLO luciferase reporter vectors containing wild and mutant 3′-UTR sequence of SNAI2 were defined as SNAI2-mutant type (SNAI2-mut) and SNAI2-wild type (SNAI2-wt), respectively. SNAI2-mut and SNAI2-wt were, respectively, cotransfected with miR-429 mimics or miR-NC into HEK-293T cells, respectively. After that, the cells were cultured for 24 hours. Finally, the luciferase activity of HEK-293T was observed by a dual-luciferase reporter assay system.

### 2.6. Tube Formation Assay

The cells treated with OGD were seed into 24-well plates coating with matrigel (BD Biosciences, San Jose, CA, USA), and cultured with DMEM containing with 15% FBS at 37°C, 5% CO_2_ for 24 hours. After that, the formation of the tubes in five random fields were observed by a microscope and photographed, and the average length of tubes in each well was measured by ImageJ software (NIH).

### 2.7. Wound Healing Assay

5 × 10^5^ HBMECs were seed into the 6-well plates and cultured for 24 hours, and the cells were transfected with related transfectants for 48 hours. After treating with OGD, the cell monolayer was scraped by 200 *μ*L pipette tip following the straight lines at the bottom of the plates, and then, exfoliated cells were washed by phosphate-buffered solution (PBS). After that, the cells were cultured with fresh serum-free DMEM for 24 hours. Finally, the healing of the cells were observed by a microscope at 0 and 24 hours, and the Image software was used to measure the width of scratches.

### 2.8. Statistical Analysis

The experiments in this study were performed at least 3 times, independently. The data were analyzed by SPSS 20.0, and the figures were charted by GraphPad Prism 8.0. Chi-squared test or ANOVA with Tukey's post hoc test was used to calculate the difference between the groups. *P* < 0.05 meant that statistical significance existed in two groups.

## 3. Results

### 3.1. MiR-429 Was Significantly Upregulated in the Serums of the Patients with Ischemic Stroke

**Figure 1:**
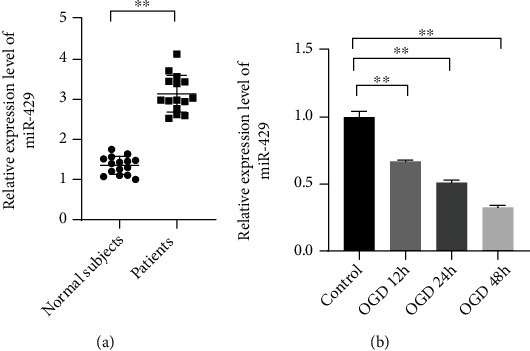
MiR-429 was significantly upregulated in the serums of patients and downregulated in OGD-induced HBMECs. (a) The relative expression level of miR-429 in the serums of the patients. (b) The relative expression level of miR-429 the HBMECs treated with OGD for 12 h, 24 h, and 48 h. ^∗∗^meant *P* < 0.01.

To analyze the connection of miR-429 and ischemic stroke, the expression levels of miR-429 in the serums of the patients and normal subjects were measured by qRT-PCR. The results showed that miR-429 was significantly upregulated in the serums of the patients compared with normal subjects (Figure 1(a), *P* < 0.01). Besides, it was found that miR-429 was significantly downregulated in OGD-induced HBMECs (Figure 1(b), *P* < 0.01).

### 3.2. MiR-429 Overexpression Regulated the Phenotype of HBMECs

**Figure 2:**
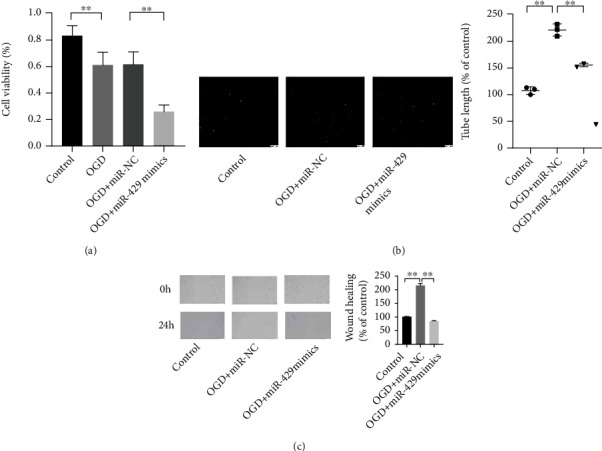
MiR-429 upregulation reduced the levels of viability, migration, and tube formation of OGD-induced HBMECs. (a) The viability of HBMECs was observed by CCK-8. (b) The angiogenesis of HBMECs was observed by tube formation assay. (c) The migration of HBMECs was observed by wound healing assay. ^∗∗^meant *P* < 0.01.

MiR-429 mimics and miR-NC were cotransfected into HBMECs before treating with OGD and reperfusion for 24 hours, in order to investigate the effects of miR-429 on the progression of ischemic stroke. CCK-8, tube formation, and wound healing were used to observe the changes in the viability and tube formation of the cells. It was found that the viability of OGD-induced cells were significantly inhibited compared with normal cells (Figure 2(a), *P* < 0.01). Compared with the OGD-induced cells transfected with miR-NC, the viability of the cells with increased miR-429 reduced significantly (Figure 2(a), *P* < 0.01). Besides, it was also found that OGD treatment could promote the tube formation and migration of HBMECs while miR-429 upregulation could inhibit this phenomenon (Figures 2(b) and 2(c), *P* < 0.01). Those observation suggests that miR-429 could inhibit the angiogenesis and migration of HBMECs after suffering ischemic injury.

### 3.3. MiR-429 Directly Targets the 3′-UTR of SNAI2

**Figure 3:**
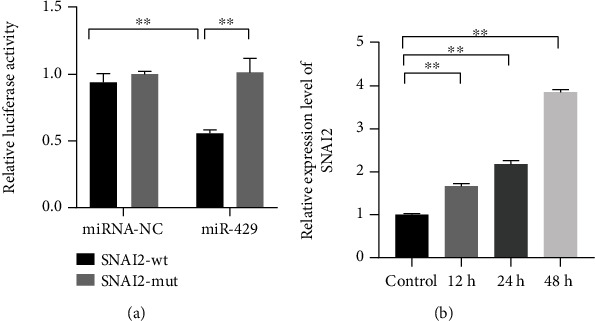
SNAI2 was a downstream target of miR-429, and SNAI2 was significantly upregulated in OGD-induced HBMECs. (a) The binding effect of miR-429 and SNAI2 was observed by dual-luciferase reporter assay. (b) The relative mRNA expression level of SNAI2 the HBMECs treated with OGD for 12 h, 24 h, and 48 h. ^∗∗^meant *P* < 0.01.

To explore the mechanism of miR-429 on the ischemic stroke, Targetscan was used for target prediction of miR-429, and dual-luciferase reporter assay was further used to confirm the binding effects of miR-429 and its targets. The results showed that SNAI2 was a downstream factor of miR-429, and miR-429 mimics showed significant effects on the luciferase activities of HEK-293T transfected with SNAI2-wt (Figure 3(a), *P* < 0.01). Furthermore, the qRT-PCR showed that SNAI2 was significantly downregulated in OGD-induced HBMECs (Figure 3(b), *P* < 0.01).

### 3.4. SNAI2 Could Reverse the Effects of miR-429 on the OGD-Induced Cells

**Figure 4:**
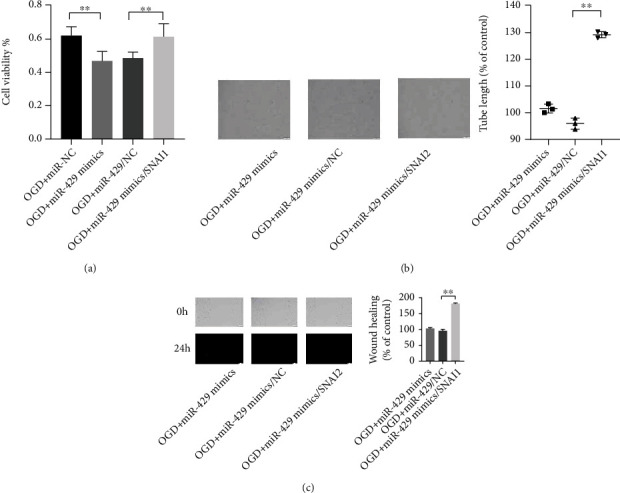
SNAI2 could reverse the effects of miR-429 on the phenotype of HBMECs treated with OGD and reperfusion. (a) The viability of HBMECs was observed by CCK-8. (b) The angiogenesis of HBMECs was observed by tube formation assay. (c) The migration of HBMECs was observed by wound healing assay. ^∗∗^meant *P* < 0.01.

Although the regulation of miR-429 on the cells has been confirmed, SNAI2 played a key role in the progression of ischemic stroke. The miR-429 mimics and SNAI2 expressed vectors were cotransfected into the HBMECs before treating with OGD and reperfusion for 24 hours to observe the phenotype change of the cells. The CCK-8 assay showed that SNAI2 significantly reversed the effects of miR-429 on the viability, migration, and tube formation of the OGD-induced cells (Figure 4).

### 3.5. MiR-429 Promotes the Progression of Ischemic Stroke via Mediating the Inactivation of GSK-3*β*/*β*-Catenin Pathway

**Figure 5:**
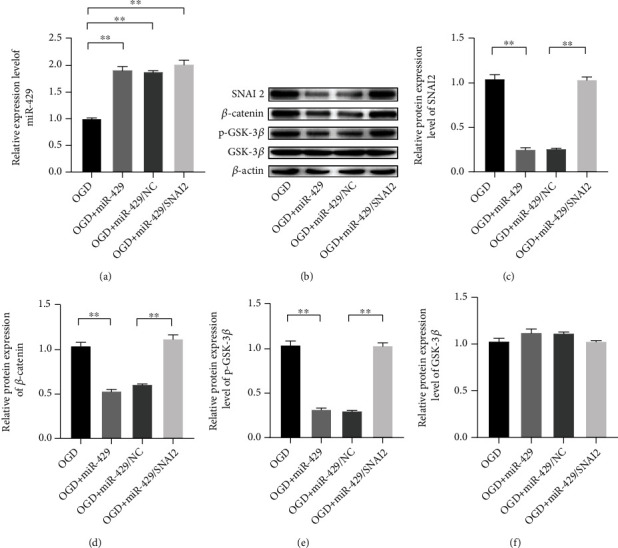
MiR-429 involved in the inactivation of GSK-3*β*/*β*-catenin via targeting SNAI2. (a) The relative expression level of SNAI2. (b–f) The protein expression levels of SNAI2, *β*-catenin, p-GSK-3*β*, and GSK-3*β*. ^∗∗^meant *P* < 0.01.

The regulation mechanism of miR-429 on the progression of ischemic stroke was further revealed by Western blot assay. The results showed that the expression levels of p-GSK-3*β* and *β*-catenin were significantly downregulated when the cells were transfected with miR-429 compared with the cells transfected with miR-NC before suffering OGD treatment. Moreover, it was found that the inhibited effects of miR-429 on activity of GSK-3*β*/*β*-catenin pathway could be rescued by SNAI2 (Figure 5).

## 4. Discussion

Ischemic stroke is one of fatal diseases to threaten the health of senior people, and the failed angiogenesis of HBMECs have been confirmed as a key event of ischemic stroke appearance [15]. This study confirmed the relationship of miR-429 and ischemic stroke, found the downstream target of miR-429, and revealed the regulation of miR-429 on the activity of GSK-3*β*/*β*-catenin pathway.

Studies' dysfunctional miRNA is one of major causes for many diseases, and the obvious differences of miRNAs' profile exist in the focused and normal tissues [16, 17]. In this study, it was found that miR-429 was significantly downregulated in OGD-induced. Some studies showed that miR-429 may play a role of tumor suppressor to inhibit the proliferation, migration, and invasion of multiple tumors. The study has indicated that miR-429 is extremely downregulated in human thyroid cancer cells, and increased miR-429 can regulate the proliferation, metastasis, and apoptosis of cancer cells via restraining the expression of ZEB1 [18]. Moreover, one study has also indicated that miR-429 silence could significantly improve the brain injury and inflammation of mice with traumatic brain injury induced by lipopolysaccharide [19]. Consequently, it seemed that miR-429 might represent as a pathogenic factor in this study, and increased miR-429 could significantly block the tube formation of HBMECs and inhibit the viability and migration ability of the cells. Tube formation serves as an important protective mechanism of cell to counter the ischemic injury [20, 21]. Pedragosa et al. [22] have proved that dysfunctional angiogenesis could impair the recovery of the mice with ischemic stroke. Therefore, this study suggests that miR-429 upregulation is a key event for the progression of the ischemic stroke. In our study, it was found that miR-429 could directly target the 3′-UTR of SNAI2, and the expression level of zinc finger protein SNAI2 (SNAI2) was negatively regulated by miR-429.

The mammalian core Hippo signaling components include Ste20 family kinases Mst1 and Mst2, which are homologous to Drosophila Hippo. Mst kinases form an active complex with WW repeat scaffolding protein Salvador (Salv), also called WW45, that phosphorylates large tumor suppressor homolog (Lats) kinase. Mammals have two Lats genes, Lats1 and Lats2, which are homologous to Drosophila Warts. Lats kinases complex with Mob to phosphorylate Yap and Taz, two related transcriptional coactivators. Hippo regulates growth and progenitor genes like Sox2, Snai2, Ccdn1, Cdc20, and l-Myc in cardiomyocytes. In livers overexpressing Yap and Hippo loss-of-function mutants, expression of c-Myc and Ccdn1 is upregulated, suggesting shared mechanisms between liver and heart [23, 24]. Although apoptosis inhibitors Birc2 and Birc5 were upregulated in Salv CKO mutant hearts, apoptosis was unchanged.

Ischemic postconditioning can increase phosphorylation of the AKT downstream targets such as GSK-3*β*, and the consideration has been given that the activation of GSK-3*β*/*β*-catenin pathway engages in the cerebral recovery of the patients after suffering ischemic injury [25, 26]. In this study, the activity of GSK-3*β*/*β*-catenin pathway of OGD-induced cell was significantly inhibited when miR-429 was upregulated. miR-429 has been verified as a key factor in angiogenesis of several tumor cells. Cheng et al. have fingered out that the decreased miR-429 induced by LncRNA XIST was the key cause of glioma angiogenesis [27]. In addition, this study also found that SNAI2 acted as an intermediary for the inactivation of GSK-3*β*/*β*-catenin, and SNAI2 upregulation could rescue the effects of miR-429 on angiogenesis of HBMECs. SNAI2 is upstream target of GSK-3*β*/*β*-catenin, and SNAI2 upregulation could promote the activation of GSK-3*β*/*β*-catenin to mediate the invasion and migration of some tumor cells. One study has testified that SNAI2 silence could extremely impede the proliferation metastasis and stemness of prostate cancer cells [28]. Hence, this study suggests the inhibitory role of miR-429 in the GSK-3*β*/*β*-catenin pathway to promote the angiogenesis of HBMECs via targeting the SNAI2. However, this study has some limitations. We only emphasized the effects of miR-429 on the phenotype of HBMECs, while more direct evidences should be achieved from the experiment *in vivo*. Additionally, the relationship between angiogenesis and functional recovery has not yet been proven. However, angiogenesis might provide a suitable microenvironment to trigger axonal outgrowth and may induce neurogenesis. The use of agents and/or cell therapies that promote angiogenesis and axonal outgrowth in the ischemic periphery may be therapeutically relevant treatment strategies.

## 5. Conclusion

This study confirmed the inhibitory effects of miR-429 on the viability, migration and tube formation of HBMECs, and revealed the downstream factor and regulation mechanism of miR-429 in the angiogenesis of the cells. In conclusion, it suggests that miR-429 could inhibit the angiogenesis of HBMECs via targeting the SNAI2.

## Figures and Tables

**Table 1:** Primer sequences of miR-429 and U6.

Name of primer	Sequences
miR-429-F	5′-AGGTCT CTGAGGGTCAAGCA-3′
miR-429-R	5′-CTGGTTGAAAAGCATGAGCA-3′
SNAI2-F	5′-TGCGATGCCCAGTCTAGAAA-3′
SNAI2-R	5′-GTGTCCTTGAAGCAACCAGG-3′
U6-F	5′-CTCGCTTCGGCAGCACA-3′
U6-R	5′-AACGCTTCACGAATTTGCGT-3′

## Data Availability

All the experimental data can be accessed by e-mail to the corresponding author.
